# Autumnal Diseases

**Published:** 1878-09

**Authors:** 


### HALL'S JOURNAL OF HEALTH.

E. H. GIBBS, A.M., M.D., Editor,

139 East Eighth Street (near Broadway), New York.

Vol. XXV.] September, 1878. [No. 9

### AUTUMNAL DISEASES.

These are chiefly diarrhæa, dysentery, and various grades of fevers, from slight "creeps" to congestive chills, for fever is the reaction of coldness, but when there is not power enough in the system to react from the cold stage, death is certain, as in congestive chill, in which the blood becomes so cold, so thick, and so impure, that it ceases to circulate, becomes stagnate, and the machinery of life stops forever. Hundreds of thousands die every autumn of the three forms of disease mentioned, but not one need die, they are avoidable diseases, their causes being known, and all that is required is to bring a very moderate amount of intelllgence to bear in avoiding these causes.

Unfortunately, the masses are either ignorant or inconsiderate; and this is the cause of nine-tenths of the sickness that exists; and neglect and improper treatment are responsible for most of the resulting maladies and the premature deaths. These diseases, like many others, come from a torpid condition of the liver, and will always yield to proper treatment where no serious complications exist. I am opposed to the administration of quinine in these cases, and only employ it as a tonic at a later period in cases of great debility. This has only occurred in my experience in one case out of about twenty. I am disposed to attribute many of the complications which often follow fever-and-ague to the unrestricted use of this drug.

The cause of Autumnal diseases is an emanation from the surface of the earth in those localities where are found in combination, heat, moisture, and vegetable matter, such as leaves, wood, etc., for the heat of eighty degrees combined with moisture induces decay, and from this decaying substance something arises which if breathed or otherwise taken into the system induces the diseases mentioned, sooner or later.

What this emanation is, has hitherto been merely a conjecture because it was so impalpable, so like thin air, that the atmosphere which contained it when subjected to chemical analysis yielded nothing beyond the constituents of pure air. But within a year or two it has been ascertained that if a quantity of air of a miasmatic locality is bottled up and is conveyed to a sleeping apartment, the person who breathes it will, in a short time, have more or less decided symptoms of fever and ague; and on examining his saliva or the inside of his mouth a living, moving thing is clearly visible with microscopic aid. Observation and experiment have shown incontrovertibly that there are two ways of escaping the ill effects of having these living things introduced in the system,—persons must avoid living in localities where the land is rich, flat and moist, or they must drain those lands; but it is possible to live in such places and have reasonbly good health simplv by keeping in the house of mornings, with a brisk blazing fire until breakfast is eaten, and take supper at sundown, because it has been found that these emanations are more poisonous at sunrise and sunset and that if the stomach is excited to action by the process of digestion the emanation is rendered innocuous, perhaps from the fact, in part, that the juices of the stomach at the time of digestion are of a character to destroy the life of these living things; but the fact remains the same, whether this supposition is true or not.

A practical use may be made of this subject in the light of these facts, in reference to breathing night air. Very many advocate the raising of windows in a sleeping apartment summer and winter, all the year round; the theory seems a good one, but experience will not corroborate it. Persons living on water courses where the "bottom lands," as they are called, are rich, luxuriant and damp will save health and life itself by keeping all outside doors and windows opening into chambers closed from sundown to sunrise during the three Autumnal months, in fever and ague or intermittent localities.

